# Performance of dual-layer spectrum CT virtual monoenergetic images to assess early rectal adenocarcinoma T-stage: comparison with MR

**DOI:** 10.1186/s13244-023-01593-5

**Published:** 2024-01-17

**Authors:** Ziqi Jia, Lei Guo, WenJing Yuan, JianHao Dai, JianYe Lu, ZhiQiang Li, Xiaohua Du, Weicui Chen, Xian Liu

**Affiliations:** 1 Department of Radiology, The Second Affiliated Hospital of Guangzhou University of Chinese Medicine, Guangzhou, China; 2 Department of Pathology, The Second Affiliated Hospital of Guangzhou University of Chinese Medicine, Guangzhou, China

**Keywords:** Virtual monochromatic imaging, Dual-layer spectral detector CT, Early rectal adenocarcinoma, Tumor staging, Magnetic resonance imaging

## Abstract

**Objectives:**

To evaluate the image quality and utility of virtual monoenergetic images (VMI) of dual-layer spectrum computed tomography (DLSCT) in assessing preoperative T-stage for early rectal adenocarcinoma (ERA).

**Methods:**

This retrospective study included 67 ERA patients (mean age 62 ± 11.1 years) who underwent DLSCT and MR examination. VMI 40–200 keV and poly energetic image (PEI) were reconstructed. The image noise, signal-to-noise ratio (SNR), contrast-to-noise ratio (CNR), and tumor contrast of different energy levels were calculated and compared, respectively. Two radiologists independently assess the image quality of the VMIs and PEI using 5-point scales. The diagnostic accuracies of DLSCT and HR-MRI for ERA T-staging were evaluated and compared.

**Results:**

The maximum noise was observed at VMI 40 keV, and noise at VMI 40–200 keV in the arterial and venous phases showed no significant difference (all *p* > 0.05). The highest SNR and CNR were obtained at VMI 40 keV, significantly greater than other energy levels and PEI (all *p* < 0.05). Tumor contrast was more evident than PEI at 40–100 keV in the arterial phase and at 40 keV in the venous phase (all *p* < 0.05). When compared with PEI, VMI 40 keV yielded the highest scores for overall image quality, tumor visibility, and tumor margin delineation, especially in the venous phase (*p* < 0.05). The overall diagnostic accuracy of DLSCT and HR-MRI for T-stage was 65.67 and 71.64% and showed no significant difference (*p* > 0.05).

**Conclusions:**

VMI 40 keV improves image quality and accuracy in identifying lesions, providing better diagnostic information for ERA staging.

**Critical relevance statement:**

Low-keV VMI from DLSCT can improve tumor staging accuracy for early rectal carcinoma, helping guide surgical intervention decisions, and has shed new light on the potential breakthroughs of assessing preoperative T-stage in RC.

**Keypoints:**

• Compared with PEI, low-keV VIM derived from DLSCT, particularly at the 40 keV, significantly enhanced the objective and subjective image quality of ERA.

• Using VMI 40 keV helped increase lesion detectability, leading to improved diagnostic accuracy for ERA.

• Low-keV VMI from DLSCT has shed new light on the potential breakthroughs of assessing preoperative T-stage in RC.

**Graphical Abstract:**

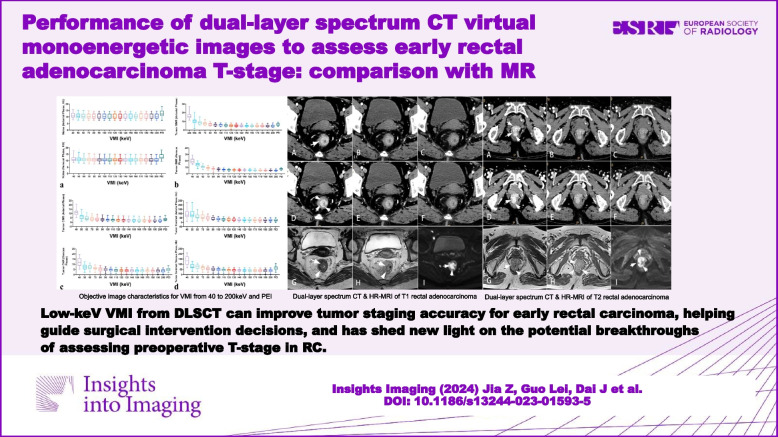

**Supplementary Information:**

The online version contains supplementary material available at 10.1186/s13244-023-01593-5.

## Introduction

Colorectal cancer (CRC) is the third most prevalent form of cancer and the third most common cause of cancer-related deaths [1]. Rectal cancer (RC) accounts for one-third of all CRC cases. Early rectal cancer (ERC) is categorized as stage I, where the tumor grows through the muscularis mucosa into the submucosa (T1) or muscularis propria (T2) without the involvement of the lymph nodes (N0) or distant metastasis (M0) [2]. The relative incidence of ERC has increased since the implementation of gastrointestinal screening programs [1]. Although total mesorectal excision (TME) remains the standard procedure for most rectal cancer patients, the rising occurrence of early-stage cancer has emphasized the significance of local excision (LE) and organ preservation as viable treatment options for these patients [3, 4]. These options can be pursued without compromising anorectal function. Therefore, precise assessment of the T-staging is crucial in informing therapeutic decision-making for RC patients.

Currently, several imaging modalities are available for evaluating the preoperative T-staging of RC, including endorectal ultrasonography (ERUS) and high-resolution magnetic resonance imaging (HR-MRI). ERUS is effective for evaluating early-stage of low and middle-rectal cancer [5]. However, its performance for evaluating intramural T2 lesions is unsatisfactory, which are often overstage with a low sensitivity (30%) [6–8]. Furthermore, ERUS is operator dependent and not applicable in stenosing lesions. HR-MRI is recommended by the National Comprehensive Cancer Network** (**NCCN) Clinical Practice Guidelines due to its superior soft tissue visualization [9]. Evaluation of mrT-staging is mainly based on the depth of tumor penetration into the rectal wall and extramural spread into the mesorectum and adjacent structures, which is largely performed with T2-weighted imaging. Nevertheless, the limitation of HR-MRI is its unreliable distinction between T1 and T2 tumors and the overstaging of T2 tumors as T3 tumors [10, 11]. Sensitivity in MRI resulted 58% in T1 and 30% in T2 cases, respectively [8]. Meanwhile, this strategy is associated with a longer waiting time and higher expenditure. Although computed tomography (CT) cannot replace ERUS and HR-MRI in T category, it has an important role in evaluating distant metastases. Therefore, CT is still advocated as a routine diagnostic modality to evaluate RC staging before treatment, stage recurrent disease, and postoperative monitoring by many current guidelines [12].

Dual-energy CT (DECT) provides quantitative information about tissue composition and improves lesion visualization. For instance, the visualization of abnormal enhanced bowel wall (ischemic small bowel and inflammatory bowel disease) has been improved by using low-keV images and iodine maps of DECT [13–17]. Studies have also demonstrated DECT is effective in identifying low-enhanced abnormalities like hypovascular liver lesions and pancreatic adenocarcinoma [18–21]. However, some limitations remain, such as artifacts caused by cross-scattered radiation in dual-source CT, misregistration between high and low voltage data, and fast kV switching waveform resembles a size wave rather than a squared wave, which leads to poor spectrum separation [22, 23]. Dual-layer spectral detector CT (DLSCT), which uses two detector layers and an X-ray tube, has recently emerged as a novel and promising imaging modality for radiological evaluation [24]. Compared to dual-energy or fast kilovoltage switching techniques, the benefit of DLSCT is that the two measurement projection data are perfectly matched in temporal and spatial co-registration, and energy spectrum scanning is realized simultaneously as conventional scanning without any additional steps or radiation agents [24]. DLSCT can also provide quantitative information, reduce photon noise and beam-hardening effects, and improve CT image quality and lesion visualization. Our previous results demonstrated that quantitative parameters derived from DLSCT could be used to assess the pT stage and histological differentiation in patients with colorectal adenocarcinoma [25]. Taguchi et al. showed that virtual monochromatic imaging (VMI) could significantly improve fecal-tagged CT colonography image quality and enhance electronic cleansing performance when using DLSCT [26]. However, there is currently little research on the clinical utility of DLSCT for evaluating the tumor stage of early rectal adenocarcinoma. We hypothesized that DLSCT VMI could improve the accuracy of local tumor staging for ERC by increasing tissue differentiation based on material decomposition. Therefore, the purpose of this study was twofold. Firstly, to investigate and compare image quality of DLSCT-derived VMI with PEI in the patients with ERA. Secondly, to evaluate DLSCT VMI would improve diagnostic accuracy of T-staging compared to HR-MRI.

## Materials and methods

### Participants

Between May 2021 and March 2023, 82 patients with pathologically confirmed early rectal adenocarcinoma (ERA) were retrospectively enrolled in this study. The review board of our hospital approved the study design. The inclusion criteria were as follows: (1) histopathologically confirmed ERA, including pT and pN stage and histological grade; (2) complete clinical information; and (3) all patients underwent DLSCT and HR-MRI examination within a week before surgery with time interval between MRI/CT examination and surgery of < 2 weeks. The exclusion criteria were as follows: (1) severe allergy to the iodinated contrast agent, impaired kidney function (creatinine clearance less than 40 ml/min/1.73 m^2^); and (2) poor image quality or severe artifacts on MR images.

### Imaging protocol

CT scanning was performed using a IQon Spectral CT (Philips Healthcare, Best, The Netherlands). The CT scan range was from the diaphragm to the symphysis pubis. DLSCT scan parameters were the following: tube voltage, 120 kV; tube current, automated exposure modulation; detection collimation, 64 × 0.625 mm; rotation time, 0.4 s; and helical pitch, 1.1. For contrast-enhanced CT scans, a body-weight-adapted dose of nonionic iodine contrast agent (ultrafast 370, Bayer Healthcare, 1.5 ml/kg body weight) was administered via the antecubital vein with a constant flow of 2.5 ml/s. Bolus tracking was performed using a region of interest within the abdominal aorta, and arterial phase (AP) acquisition was started when the attenuation reached a predefined threshold of 150 Hounsfield units (HU). Venous-phase (VP) images were acquired 40 s after AP.

Conventional and quantitative spectral images were reconstructed and analyzed using post-processing software (IntelliSpace Portal Version 10.0, Philips Healthcare). A VMI of 40–200 keV was reconstructed using a reconstruction algorithm (Spectral Level 3, Philips Healthcare) with a 10-keV interval. The PEI was reconstructed using an iterative reconstruction algorithm (iDose4, Philips Healthcare) with a constant standard soft tissue kernel. All images were reconstructed at a slice thickness of 2 mm for further analysis.

The patient examination protocol for the CT scanner included the mean mAs and dose index of the entire examination. These values were documented to ensure accurate and thorough recording.

Rectal MR was conducted using various 3.0 T MR scanners from Siemens Healthcare (Prisma, Verio) and Philips Healthcare (Ingenia). MR sequences included T1WI, high-resolution T2WI, T1-weighted enhanced images (with Magnevist®, Bayer HealthCare, Guangzhou, China, at a dose of 0.2 ml/kg of body weight), and conventional DWI (*b* = 0, 1000 s/mm^2^). The detailed parameters of the MR protocol are listed in Table S1.

### DLSCT image objective analysis

Two radiologists (with 25 and 16 years of experience in gastrointestinal radiology), blinded to the clinical and pathological information, independently evaluated the DLSCT data and performed an objective analysis.

Regions of interest (ROIs) were drawn manually to measure the mean attenuation of the tumor (HU _tumor_) and normal intestinal wall tissue (HU _intestinal wall_) on –40–200 keV VMI and PEI images in the arterial and venous phases. The ROI was drawn by selecting identical axial sections where the maximum diameter of the lesion was located and covering the lesion as much as possible, avoiding fat and necrosis. Each ROI was kept in a consistent position and size on all sets of VMIs and PEI in both the arterial and venous phases and measured twice to ensure consistency with the averaged values used for the analysis. The normal intestinal wall was delineated into the upper and lower layers adjacent to the tumor. Image noise was defined as the standard deviation within the muscle (SD _muscle_) in the same slice of 10–15 mm^2^ to eliminate selective disparities. The signal-to-noise ratio (SNR) and contrast-to-noise ratio (CNR) of rectal tumors and tumor contrast were calculated using the following formulas:

$$\textrm{SNR}={\textrm{HU}}_{\textrm{tumor}}/{\textrm{SD}}_{\textrm{muscle}}$$

$$\textrm{CNR}=\left({\textrm{HU}}_{\textrm{tumor}}-{\textrm{HU}}_{\textrm{intestinal}\ \textrm{wall}}\right)/{\textrm{SD}}_{\textrm{muscle}}$$

$$\textrm{Tumor}\ \textrm{contrast}={\textrm{HU}}_{\textrm{tumor}}-{\textrm{HU}}_{\textrm{bowel}\ \textrm{wall}}$$

### DLSCT image subjective analysis

The aforementioned two radiologists reviewed the DLSCT VMI images. Radiologists used 5-point scales to assess the quality of the images, tumor visibility, and tumor margin delineation in a blinded and randomized manner. The scale ranged from 1 (unacceptable) to 5 (excellent) for overall image quality and 1 (unacceptable) to 5 (excellent) for tumor visibility and margin delineation.

### DLSCT and MRI evaluation in T-staging of ERC

All DLSCT VMIs and HR-MRI examinations were analyzed in consensus by the aforementioned two radiologists, respectively. Both of them were blind to the histopathologic examinations.

Tumor staging based on the spectrum CT and MR was separately analyzed according to the AJCC (American Joint Committee on Cancer), 8th edition. To minimize recall bias, the DLSCT and HR-MRI evaluation were conducted as two separate assignments, with a 3-month washout period in between. DLSCT T-staging was determined using objective and subjective analysis results that provide the best tumor visibility. MR T-stage evaluation was conducted based on the multiparameter MRI, including T1WI, T2WI, and DWI sequences [27].

### Histological analysis

All patients underwent surgery within 1 week after DLSCT and HR-MRI examinations. The tumor specimens were reviewed by a pathologist with 14 years of experience in gastroenterology using HE and immunohistochemical staining. TNM stages and tumor grades were determined based on AJCC, 8th edition. Early rectal tumors are T1 or T2 tumors without lymph nodes or distant metastasis (T1-2, N0, M0). T1: tumor invades the submucosa (through the muscularis mucosa, but not into the muscularis propria). T2: tumor invades the muscularis propria. Tumor grade was classified according to the percentage of gland formation, with grade 1 indicating well-differentiated tumors (> 95% gland formation), grade 2 indicating moderately differentiated tumors (50–95% gland formation), and grade 3 indicating poorly differentiated tumors (< 50% gland formation).

### Statistical analysis

Statistical analyses were performed using SPSS (version 20.0; SPSS Inc., Chicago, IL, USA) and GraphPad Prism 9.0 (GraphPad Software, San Diego, CA, USA).

The inter-reader reproducibility of the noise, HU of the lesion, and the intestinal wall was calculated using the intraclass correlation coefficient (ICC). The Kolmogorov–Smirnov test was used to assess data distribution. Continuous variables are presented as mean (standard deviation) or median (interquartile range) depending on the data distribution. The nonparametric Friedman test was used to compare quantitative variables between VMI and PEI in the arterial and venous phases. The kappa coefficient (*k*) was used to measure the inter-reader agreement of the overall image quality, tumor visibility, and delineation (*k* = 0.8–1.00 excellent; 0.61–0.8 substantial; 0.41–0.60 moderate; 0.21–0.40 fair; 0.00–0.20 poor). Subjective quality scores were compared using nonparametric tests (Kruskal–Wallis test, followed by Steel–Dwass post hoc test).

Using the pathological results as a reference, the overall accuracy, overstaging, and under-staging rates of DLSCT and MR were calculated. The chi-square test was used to compare the diagnostic performances of DLSCT and HR-MRI. A *p*-value less than 0.05 was considered statistically.

## Results

### Participants

**Fig. 1:**
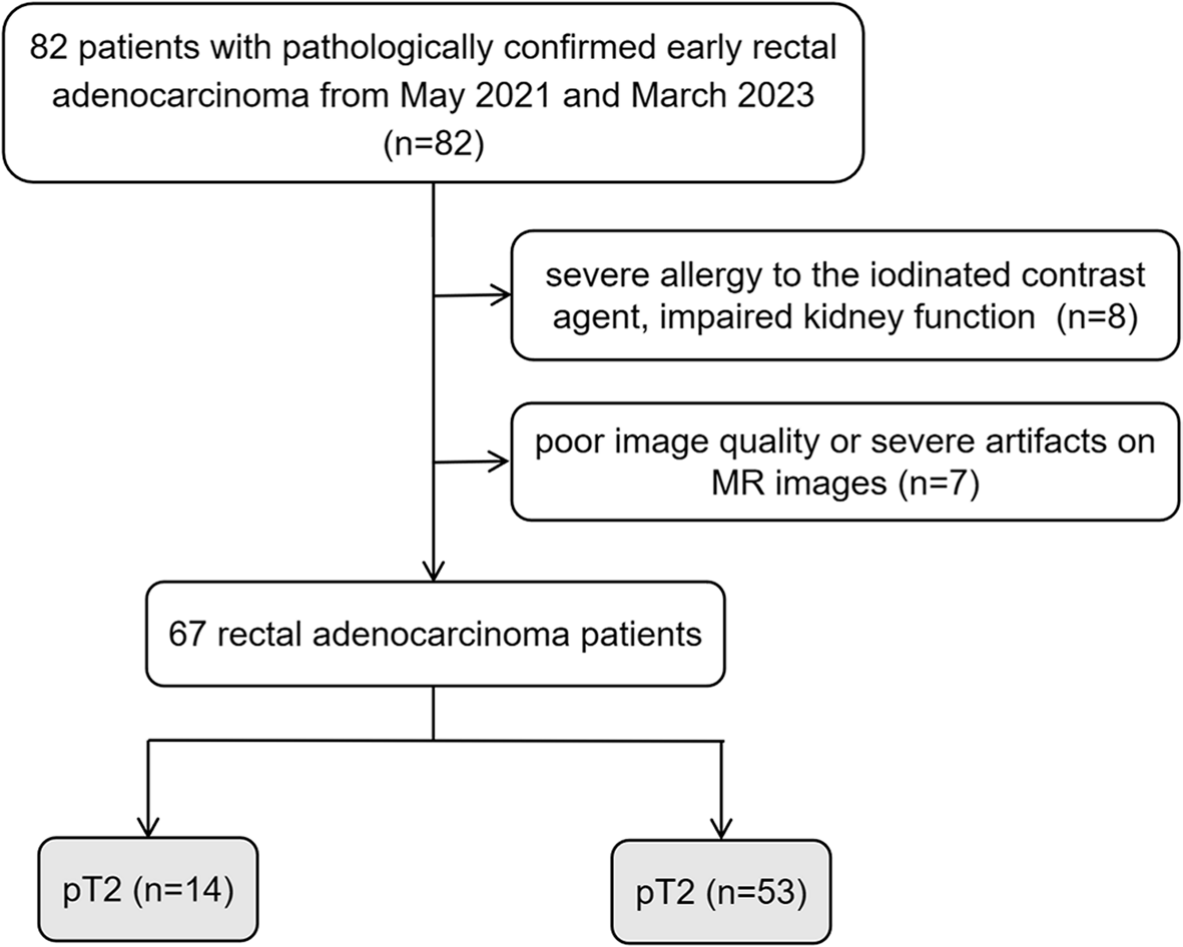
Flowchart shows patient selection

In total, 67 pathologically confirmed early rectal adenocarcinoma patients were enrolled in the study, consisting of 25 females and 42 males (mean age, 62 ± 11.1 years) (Fig. 1). Detailed patient demographics are shown in Table 1.

**Table 1:** Demographic characteristics of the patients

Characteristic	Number (%)
Gender	
Male	25 (37.31%)
Female	42 (62.69%)
Age (years)	
Mean ± SD	62 ± 11.1
pT Stage	
T1	14 (20.90%)
T2	53 (79.10%)
pN stage	
N0	67 (100%)
Grade	
G1	5 (7.46%)
G2	56 (83.58%)
G3	6 (8.96%)
Tumor Location	
Upper	5 (7.46%)
Middle	56 (83.58%)
Lower	6 (8.96%)
Treatment	
TEM	24 (35.82%)
EMR/ESD	11 (16.42%)
TME	32 (47.76%)

Data are shown in number (%) or mean ± standard deviation

*EMR* endoscopic mucosal resection, *ESD* endoscopic submucosal dissection, *TEM* transanal endoscopic resection, *TME* total mesorectal excision

### Objective image quality

Inter-observer agreement was excellent (intraclass coefficient > 0.90) for all the variables. The ICCs ranged from 0.900 to 0.995 for HU tumor, HU intestinal wall, and SD muscle of VMI 40–200 keV and PEI in the arterial and venous phases. Details of the ICCs values are provided in Table S2.

**Fig. 2:**
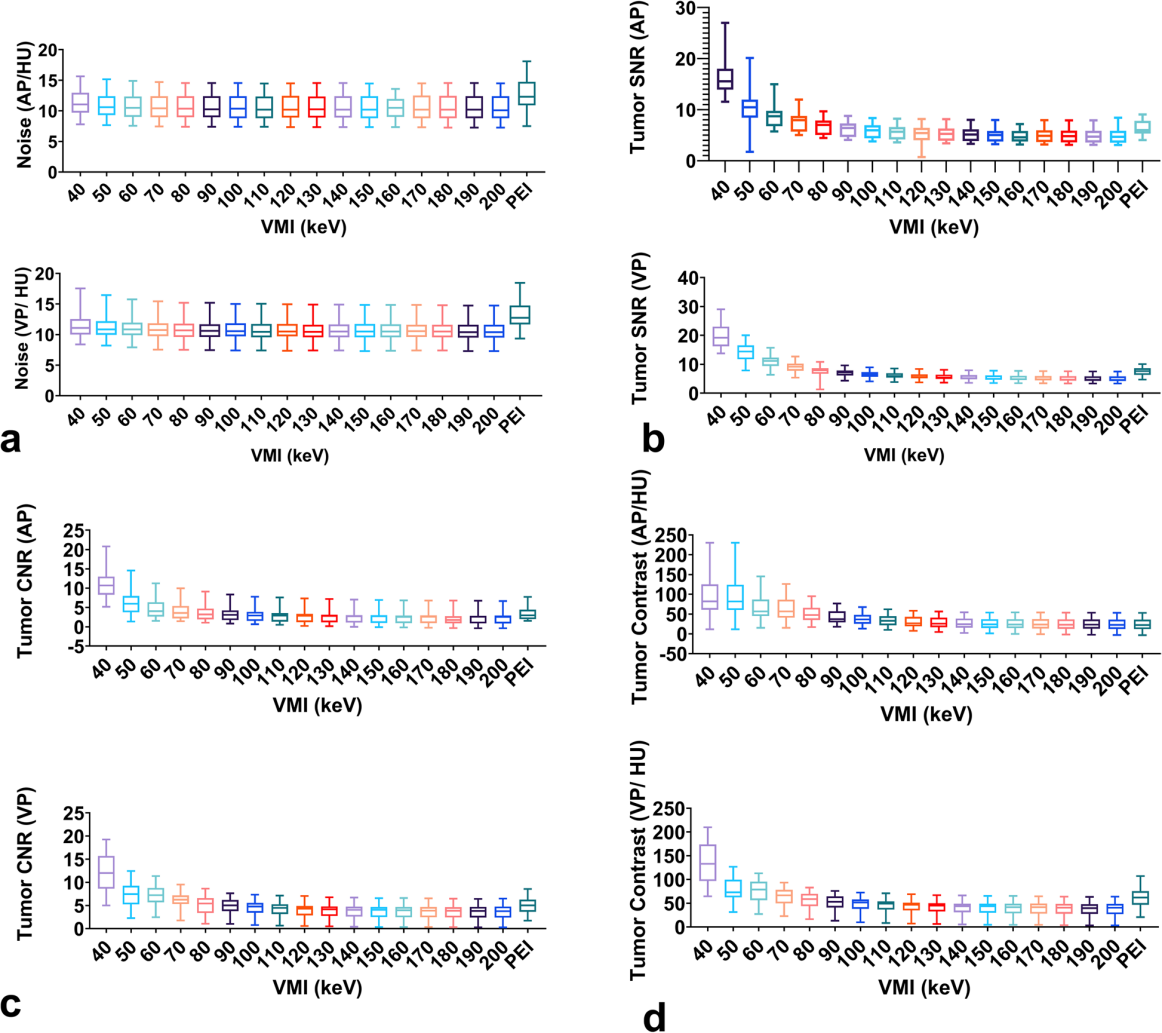
Objective image characteristics for VMI from 40 to 200 keV and PEI. The data for image noise (**a**), tumor SNR (**b**), tumor CNR (**c**), and tumor contrast (**d**) at VMI 40–200 keV and PEI in the arterial and venous phases are presented using box-and-whisker plots. Each box in the plot shows the upper and lower quartiles of the data, while the median value is displayed as a horizontal line

The noise levels in the arterial and venous phases were low at all VMI energy levels. The maximum noise was observed at 40 keV [AP 11.05 (9.70, 12.95); VP 11.10 (10.00, 12.55)], followed by a slight decrease with increased energy levels, and the noise was relatively stable at all the energy levels. Multiple comparisons demonstrated that the noise at 50–200 keV was significantly lower than that of PEI in the AP (all *p* < 0.05) and that the noise at all energy levels was significantly lower than that of PEI in the VP (all *p* < 0.05). Noise at 40–200 keV in the arterial and venous phases showed no significant differences (all *p* > 0.99) (Fig. 2a).

The tumor SNR and CNR in the arterial and venous phases are shown in Table 2. As the energy level decreased, the SNR and CNR gradually increased. The VMI 40 keV provided the highest tumor SNR [15.56 (13.93, 18.00)] in the AP, although there was no statistical difference between 40 and 50 keV [10.48 (8.43, 11.97)] (*p* > 0.99, Fig. 2a). Tumor SNR of VMI 40–60 keV was significantly higher than that of PEI [15.56 (13.93, 18.00)–8.70 (6.76, 9.78) vs. 6.00 (5.31, 7.82), *p* < 0.05], while tumor SNR of VMI 130–200 keV was significantly lower than that of PEI [5.26 (3.91, 6.30)–4.68 (3.52, 5.87), *p* < 0.05]. In the VP, 40 keV achieved the maximum tumor SNR [19.21 (16.25, 23.00)], while tumor SNR at the low energy levels showed no difference between 40, 50, and 60 keV (all *p* > 0.99) (Fig. 2b).

**Table 2:** Subjective image quality analysis

	Image Quality	Tumor Visibility	Tumor Delineation
VMI 40 keV			
Arterial Phase	4.24 ± 0.43*	4.21 ± 0.51*	4.16 ± 0.57*
Venous Phase	4.28 ± 0.45*	4.25 ± 0.49*	4.26 ± 0.17*
VMI 50 keV			
Arterial Phase	3.96 ± 0.51	3.80 ± 0.53	3.70 ± 0.55
Venous Phase	3.92 ± 0.47	3.71 ± 0.54	3.67 ± 0.56
VMI 60 keV			
Arterial Phase	3.71 ± 0.54	3.67 ± 0.53	3.58 ± 0.50
Venous Phase	3.72 ± 0.55	3.62 ± 0.51	3.52 ± 0.53
PEI			
Arterial Phase	2.18 ± 0.67	2.25 ± 0.61	2.22 ± 0.57
Venous Phase	2.25 ± 0.64	2.22 ± 0.60	2.19 ± 0.56

Data are shown in mean ± standard deviation

*VMI* virtual monochromatic imaging, *PEI* polyenergetic image

* indicates significant differences (*p* < 0.05) of VMI 40–60 keV compared to PEI

The tumor CNR ranged from [10.71 (8.34, 13.00), 40 keV] to [2.53 (0.86, 2.91), 200 keV] and from [12.00 (8.62, 15.69), 40 keV] to [3.77 (2.39, 4.74), 200 keV] for the arterial and venous phases, respectively. The highest CNR was obtained at 40 keV [10.71 (8.34, 13.00), AP; 12.00 (8.62, 15.69), VP], which was significantly greater than that at the other energy levels and PEI (all *p* < 0.05) (Fig. 2c).

**Fig. 3:**
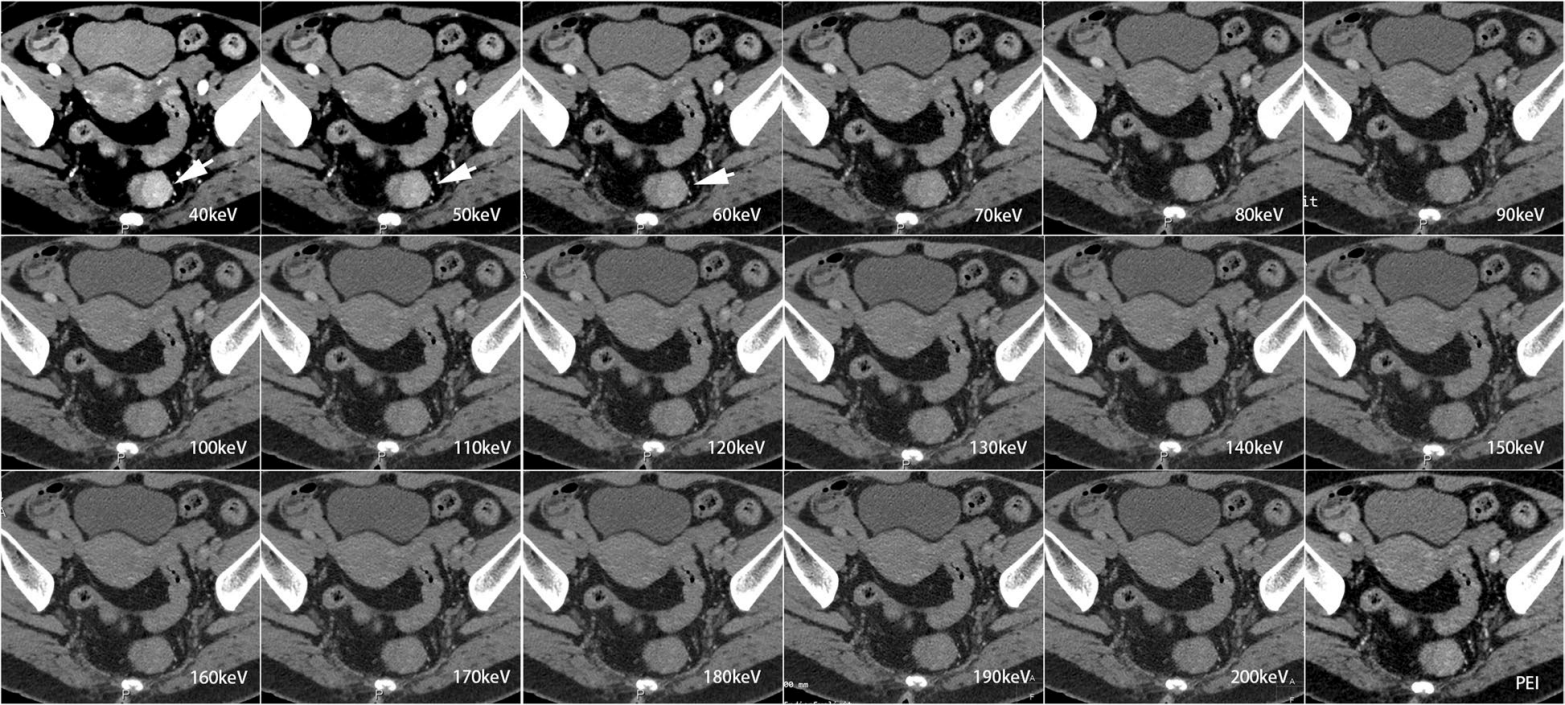
Contrast-enhanced axial arterial phase in 66-year-old female with T2 stage rectal adenocarcinoma. VMI 40–200 keV of arterial phase shows that tumor SNR, tumor CNR, and tumor contrast increases as the energy level decreases. The highest SNR, CNR, and tumor contrast were obtained on the VMI 40 keV, followed by 50 and 60 keV. There was no difference in the SNR, tumor contrast among VMI 40, 50, and 60 keV. The best image quality, tumor visibility, and tumor delineation were obtained at VMI 40 keV

**Fig. 4:**
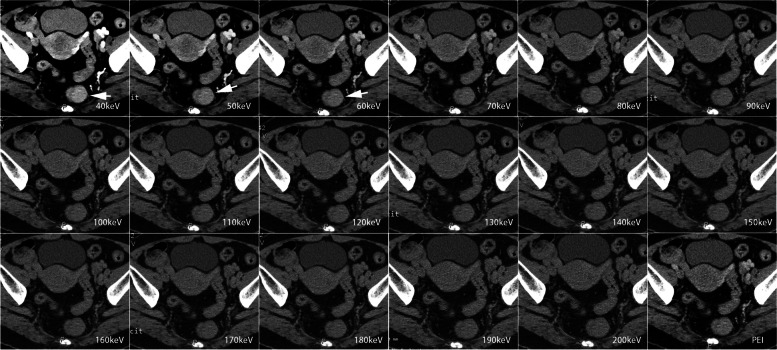
Contrast-enhanced axial venous phase in the same patient in Fig. 3. VMI 40–60 keV outperformed PEI in objective analysis and subjective scores. The highest SNR, CNR, and tumor contrast were obtained on the VMI 40 keV, which were significantly higher than other energy levels and PEI (all *p* < 0.05). VMI 40 keV in the venous phase yielded the highest overall image quality, tumor visibility, and tumor margin delineation scores

Tumor contrast gradually increased as the monoenergetic level decreased in both the arterial and venous phases. The maximum tumor contrast in the AP was obtained at 40 keV, but it did not show a significant difference when compared to that at 50 and 60 keV (Figs. 2d and 3). In the VP, the tumor contrast at 40 keV reached the maximum value, which was significantly higher than that at the other energy levels and PEI (all *p* < 0.05) (Figs. 2d and 4). The detailed data of objective image quality analysis was shown in Table S3.

### Subjective image quality

Based on the objective analysis results, a subjective analysis was performed using VMI 40–60 keV and PEI. The agreement between the overall image quality, tumor visibility, and tumor margin delineation ranged from substantial to excellent (kappa value = 0.688, 0.897, and 0.763 in the AP and 0.688, 0.897, and 0.763 in the VP), indicating good inter-observer reliability. VMI 40–60 keV outperformed PEI in all criteria as per the subjective scores. VMI of 40 keV yielded the highest scores for overall image quality, tumor visibility, and tumor margin delineation, especially in the VP (*p* < 0.001). Table 3 summarizes the results of subjective analyses.

**Table 3:** Performance of DLSCT and MRI for staging pT1 and pT2 rectal cancers

	Accuracy (95%CI)	Overstaged Ratio	Understaged Ratio	Sensitivity	Specificity
DLSCT					
pT1 (*n* = 14)	50.00% (0.218–0.782)	0%	50.00%	42.86%	88.68%
pT2 (*n* = 53)	82.60% (0.716–0.936)	17.40%	0%	71.70%	42.86%
Total	65.67% (0.543–0.771)	25.37%	8.96%	57.28%	65.77%
MRI					
pT1 (*n* = 14)	61.54% (0.352–0.876)	0%	38.46%	57.14%	90.57%
pT2 (*n* = 53)	87.00% (0.772–0.967)	13.00%	0%	75.47%	57.14%
Total	71.64% (0.609–0.824)	20.9%	7.46%	66.31%	73.86%

Data are shown in number (%)

*DLSCT* dual-layer spectrum CT, *MRI* magnetic resonance imaging

### Comparison of diagnostic accuracy for T-staging between DLSCT and HR-MRI

**Fig. 5:**
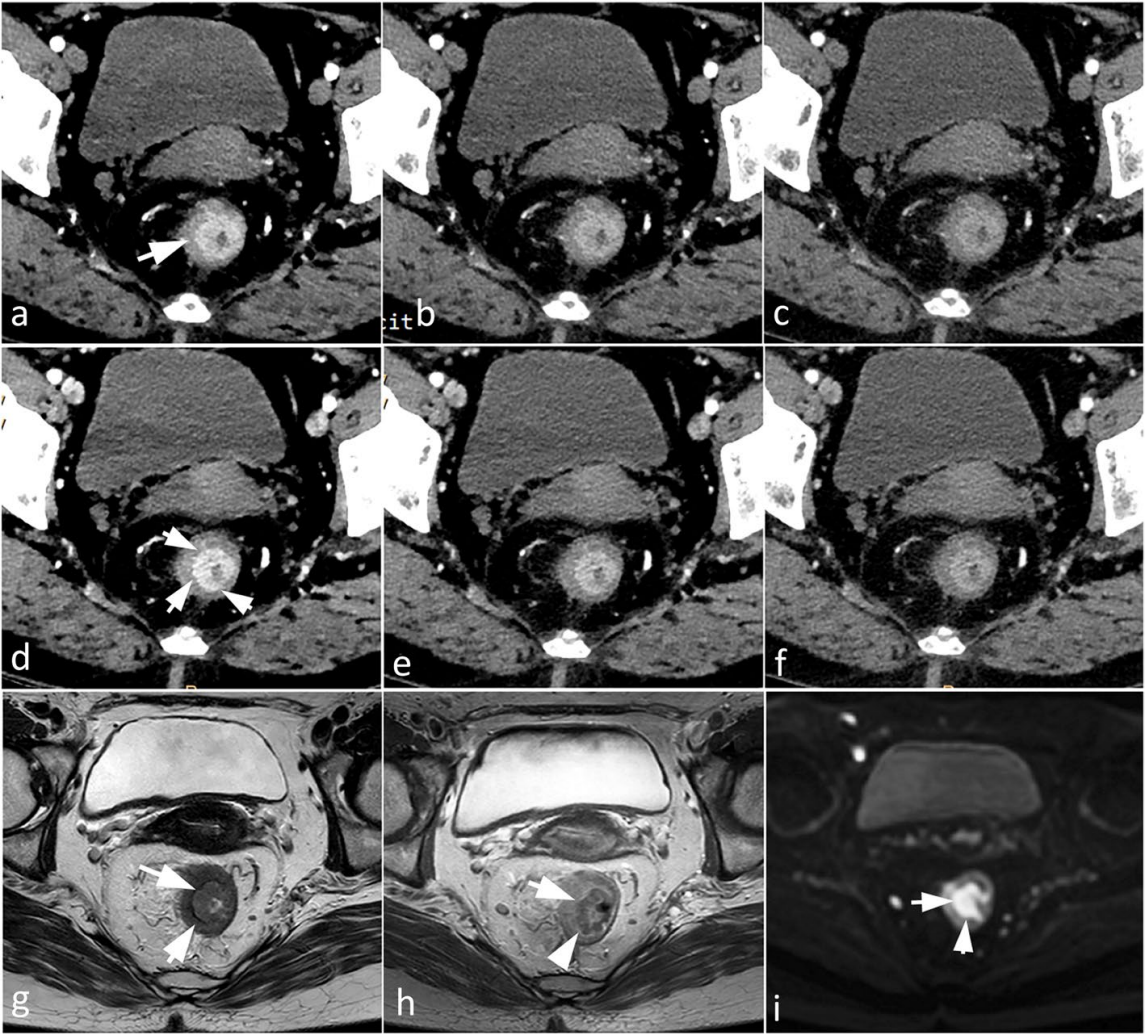
A 59-year-old male with T1 rectal adenocarcinoma who underwent dual-layer spectral CT and HR-MRI. VMI 40–60 keV in the arterial phase (**a**–**c**) and venous phase (**d,e**) demonstrate that the tumor is confined to the submucosa. The VMI 40 keV of venous phase (**d**) provides the best tumor conspicuity and margin delineation and exhibits no significant difference compared to MRI (**g**: axial T2WI; **h**: Gd-DPTA enhanced T1WI; **i**: DWI at *b* = 1000 s/mmm^2^)

**Fig. 6:**
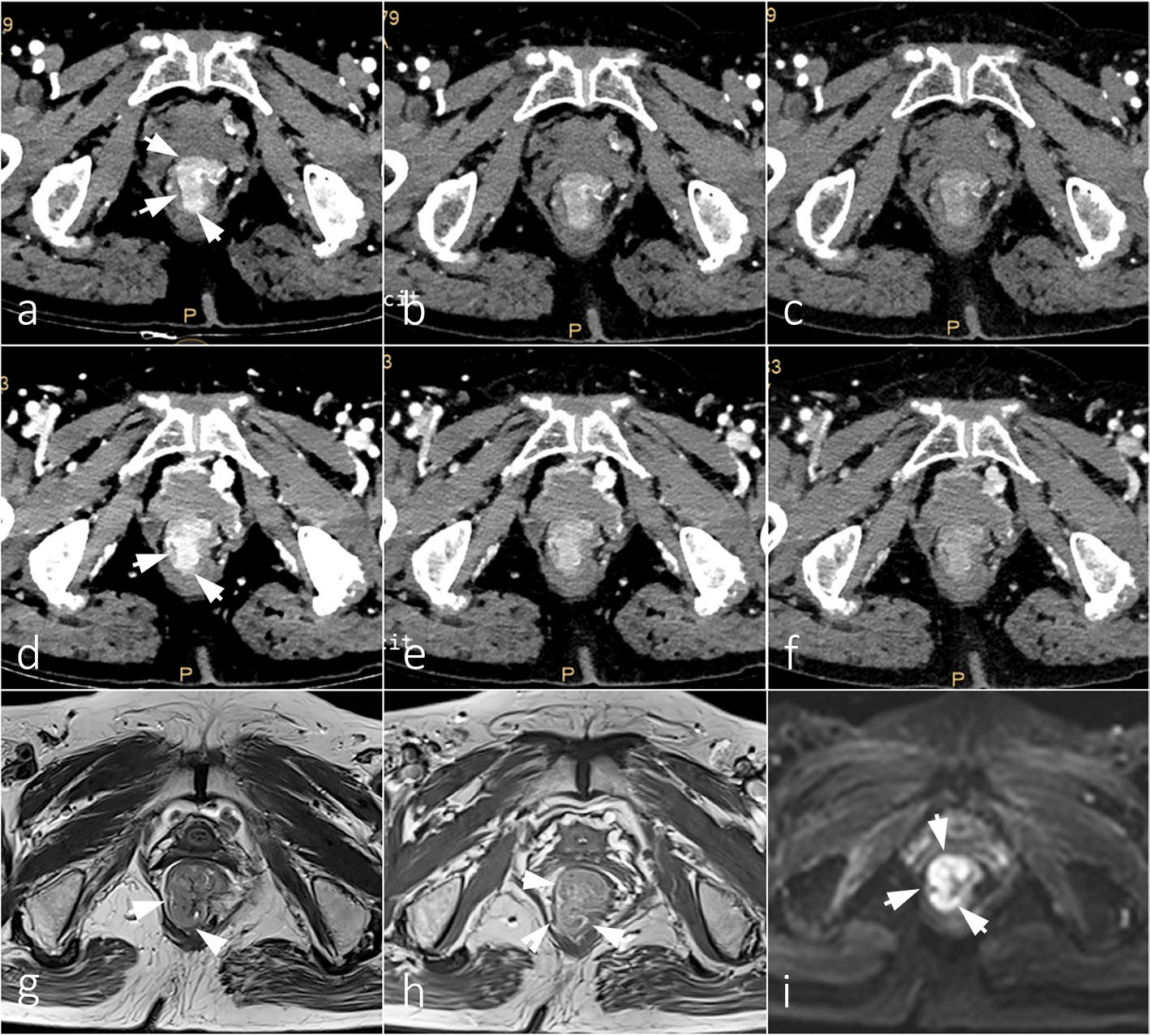
A 76-year-old female with T2 rectal adenocarcinoma who underwent dual-layer spectral CT and HR-MRI. VMI 40–60 keV in the arterial phase (**a**–**c**) and venous phase (**d,e**) demonstrate that the tumor has grown into the muscularis propria and has not yet broken through the serosal layer. The VMI 40 keV of venous phase (**d**) provides the same capability of stage evaluation as MRI (**g**: axial T2WI; **H**: Gd-DPTA enhanced T1WI; **i**: DWI at *b* = 1000 s/mmm^2^)

According to the objective and subjective analysis results, a VMI 40–60 keV of arterial and venous phases was selected to assess the T-stage. The diagnostic accuracies of DLSCT and HR-MRI for T-staging were evaluated and compared. Inter-observer agreement in DLSCT and MRI T-stage was significant (DLSCT: kappa value = 0. 82, *p* < 0.001; MRI: kappa value = 0.75, *p* < 0.001). For ERA T-stage, the overall DLSCT diagnostic accuracy was 65.67% (44/67, 57.28% sensitivity and 65.77% specificity, 95% confidence interval 54.3 to 77.1%), with six pT2 patients being underdiagnosed with T1, eight pT1 patients overdiagnosed with T2, and nine pT2 patients overdiagnosed with T3. The diagnostic accuracy of HR-MRI was 71.64% (48/67, 66.31% sensitivity and 73.86% specificity, 95% confidence interval 60.9 to 82.4%), including five pT2 patients mistaken as T1, six pT1 patients misdiagnosed as T2, and eight pT2 patients inaccurately diagnosed as T3. The accuracy for T1-2 (*χ*^2^ = 0.555, *p* = 0.45), T1 (*χ*^2^ = 0.337, *p* = 0.56), and T2 staging (*χ*^2^ = 0.337, *p* = 0.56) between DLSCT and HR-MRI showed no significant differences (all *p* > 0.05). The detailed results are presented in Table 3. The representative cases are shown in Figs. 5 and 6.

## Discussion

In the current study, we evaluated the imaging quality of VMI using DLSCT in ERA and compared its T-stage evaluation capability to that of HR-MRI. Our findings indicate that when compared with PEI, low noise levels were obtained from VMI 40 to 200 keV in both the arterial and venous phases. The best image quality and lesion detectability were achieved with a VMI of 40 keV in the VP, which showed higher SNR, higher CNR, better tumor contrast, and acceptable image noise. In addition, we found that the accuracy of DLSCT was comparable to that of HR-MRI in evaluating the T-stage of ERA.

Monochromatic images are generally synthesized through basis material decomposition (projection or image domain) and known densities of the basis materials [28]. Image noise is an important criterion for evaluating image quality. Our study showed that the highest noise level was observed at 40 keV and that the noise magnitude decreased as the VMI energy increased. In the venous phase, the noise at all VMI energy levels was significantly lower than that of the PEI. This finding is consistent with the results of a previous study by Kalisz et al., who found that the VMI obtained from DLSCT had low noise across the entire energy spectra [29]. Although previous phantom studies and patient cohorts have shown high image noise in low-energy VMI [30], which limits its utility and diagnostic capabilities, we found no significant noise differences across the entire scope of VMIs, regardless of whether it was in the arterial or venous phase. This result is similar to that of a study on abdominal phantoms by Sellerer et al., which showed that DLSCT produced no significant alteration in noise throughout the entire range of VMIs [31]. This can be attributed to the perfect alignment of the low- and high-energy spectra in the projection domain, which reduces beam-hardening artifacts and anti-correlated noise [32].

We found that the tumor SNR, tumor CNR, and tumor contrast gradually increased as the energy levels decreased, and the highest tumor contrast was obtained at VMI 40 keV with a significant improvement. VMI at 40–60 keV had a higher SNR than PEI, with VMI at 40 keV being the highest. This is likely owing to the following factors. First, the higher photoelectric attenuation of the low-energy VMI image was close to the K-edge of iodine (33.17 keV), resulting in higher absorption and enhanced iodine contrast in the image [20]. Second, DLSCT provides a constant low noise across the entire spectrum of energies. Previous studies have shown that VMIs obtained from detector-based spectral CT achieved significant SNR and CNR improvements compared to conventional 120 kVp images. For instance, Lee et al. showed that VMI_40_ provided optimal CNR for abnormal and normal small bowel walls on dual-layer dual-energy CT enterography (DE-CTE) [17]. Nagayama et al. demonstrated that low-keV VMI improved the image quality and yielded adequate diagnostic tumor detectability in patients with dual pancreatic adenocarcinoma and hypovascular hepatic metastases [20]. Concordant with these findings, our subjective analysis revealed that the low VMI series performed better than did the PEI series with superior tumor quality, tumor visibility, and more prominent tumor delineation, while a VMI of 40 keV was considered optimal for improved lesion delineation and detection performance and increased readers’ confidence. Additionally, we found that the SNR, CNR, and tumor contrast in the venous phase were higher than those in the AP. This may be due to the immature neovascularization of ERC, which makes the contrast agent more susceptible to extravasation. The contrast agent is easily retained and distributed in the venous phase; therefore, it can better reflect the attenuation of iodine in tumor tissue [33].

Surgical treatment strategy for rectal cancer depends on accurate preoperative staging. Local excision is suitable only for early-stage patients with cT1N0 and low grade. For patients with cT1/T2 and negative N status, transabdominal radical resection without neoadjuvant therapy is recommended [4, 34]. MR is the preferred modality for rectal cancer staging because of its ability to evaluate the depth of tumor invasion. However, distinguishing between T1 and T2 lesions remains challenging. A recent Dutch population-based study demonstrated that 54.7% of patients with pT1 tumors were overstaged by MRI alone [35]. In our study, the depth of tumor invasion into the rectal bowel wall was accurately evaluated by using the combination of the high-contrast of VMI 40 keV and noticeable tumor enhancement. DLSCT provided 65.67% diagnostic accuracy for the T category, similar to that of MRI, and a shorter scan time could reduce the possibility of motion artifacts.

Our study had several limitations. First, it was a retrospective study with a small sample size. A prospective study with larger samples is required to verify our results. Second, we only focused on the feasibility of the VMI for the evaluation of early rectal adenocarcinoma. Other parameters obtained from dual-energy CT, such as iodine map, can improve the visibility of bowel wall hypoenhancement [14, 36]. Additionally, color-coded effective *Z*-images after enhancement were helpful in increasing the tumor conspicuity. Third, DLSCT image quality is affected by other parameters such as the reconstruction kernel, collimation, slice thickness, iodine contrast material dose, patient size, and dose level [37–39]. Notably, low-energy VMI can improve image contrast with lower amounts of iodine contrast agents and radiation doses. Further studies should focus on improving image quality and lesion detectability with reduced radiation dose and contrast optimization. Last, our study focused on the diagnostic capability of VMI in evaluating T-stage of early rectal cancer. Besides VMI, dual-energy CT can provide multiple quantitative parameters regarding tissue’s biological information and offers a non-invasive method for predicting histopathological and molecular identification [40–43]. Further research should be carried out to explore the relationship between dual-energy CT-derived parameters and genotype classification of rectal cancer.

## Conclusion

In conclusion, low-keV VIM derived from DLSCT improved early rectal adenocarcinomas’ objective and subjective image quality. VMI 40 keV of DLSCT provided low image noise and high SNR, CNR, and tumor contrast, which improved the accuracy of lesion identification and provided better diagnostic information for the staging of ERA. The findings provide evidence that DLSCT has shed new light on the potential breakthroughs of assessing preoperative T-stage in RC.

### Supplementary Information

**Additional file 1: Table S1. **MRI sequence parameters of T2WI, DWI, T1WI, and T1-CE of different MR devices. **Table S2.** Inter-observer reliability for HU _tumor_, HU _intestinal wall_ and SD _muscle_. **Table S3.** Objective image quality analysis.

## Data Availability

The data that support the findings of this study are available on request from the corresponding author. The data are not publicly available due to privacy or ethical restrictions.

## Abbreviations

CNR: Contrast-to-noise ratio
DECT: Dual-energy CT
DLSCT: Dual-layer spectrum computed tomography
ERA: Early rectal adenocarcinoma
ERUS: Endorectal ultrasonography
HR-MRI: High-resolution magnetic resonance imaging
NCCN: National Comprehensive Cancer Network
PEI: Poly energetic image
RC: Rectal carcinoma
SNR: Signal-to-noise ratio
TME: Total mesorectal excision
VMI: Virtual monoenergetic images
