# Correction: Performance of dual-layer spectrum CT virtual monoenergetic images to assess early rectal adenocarcinoma T-stage: comparison with MR

**DOI:** 10.1186/s13244-024-01772-y

**Published:** 2024-08-09

**Authors:** Ziqi Jia, Lei Guo, WenJing Yuan, JianHao Dai, JianYe Lu, ZhiQiang Li, Xiaohua Du, Weicui Chen, Xian Liu

**Affiliations:** 1 Department of Radiology, The Second Affiliated Hospital of Guangzhou University of Chinese Medicine, Guangzhou, China; 2 Department of Pathology, The Second Affiliated Hospital of Guangzhou University of Chinese Medicine, Guangzhou, China

**Correction to:** ***Insights into Imaging***

10.1186/s13244-023-01593-5, published online 17 January 2024

The original article mistakenly reports an incorrect grant number in the Funding (‘2023A03J024’); the grant number should instead read ‘2023A03J0245’.

