# Supplementary material for: Performance of dual-layer spectrum CT virtual monoenergetic images to assess early rectal adenocarcinoma T-stage: comparison with MR
Source: Insights Imaging. 2024 Jan 17;15:11. doi: 10.1186/s13244-023-01593-5 (PMC10792143; doi:10.1186/s13244-023-01593-5)
Supplement: Supplementary file 1 — Additional file 1: Table S1. MRI sequence parameters of T2WI, DWI, T1WI, and T1-CE of different MR devices. Table S2. Inter-observer reliability for HU tumor, HU intestinal wall and SD muscle. Table S3. Objective image quality analysis. [file 13244_2023_1593_MOESM1_ESM.pdf]

Performance of dual-layer spectrum CT virtual monoenergetic images to assess early rectal adenocarcinoma T-stage: comparison with MR

ELECTRONIC SUPPLEMENTARY MATERIAL

Table S1. MRI sequence parameters of T2WI, DWI, T1WI, and T1-CE of different MR devices.

	Siemens Verio 3.0T			Philips Ingenia 3.0T			Siemens Prisma 3.0T		
	T2WI	DWI	T1WI/T1-CE*	T2WI	DWI	T1WI/T1-CE*	T2WI	DWI	T1WI/T1-CE*
TR /TE(ms)	6350/93	5900/83	3.97/1.29	3664/100	3514/81	3.6/1.32	8040/89	4900/53	3.97/1.29
ETL	28	1	1	17	53	1	19	1	1
Flip Angle (°)	140	90	10	90	90	10	160	90	10
Slice thickness (mm)	3	5	4	3	4	4	3	4	4
FOV (cm)	200×200	270×360	210×210	200×200	320×320	210×210	200×200	180×320	210×210
(Pixel) Bandwidth	260	1628	1040	325	2303	1377	200	1985	1040
Imaging Frequency (Hz)	123.2	123.2	123.2	127.8	127.8	127.8	123.2	123.2	123.2
Matrix	320×256	192×115	224×216	288×228	108×106	224×216	320×240	140×104	224×216
Percent Sampling (%)	80	80	100	84	99	100	80	100	100
Number of Average	2	2	1	2	2	1	1	1	1

T1-CE: contrast-enhanced T1-weighted imaging; TR: Repetition time; TE: Echo Time; ETL: Echo Train Length; FOV: Field of View.

* indicate T1WI and T1-CE sequences parameters of axial section.

Table S2. Inter-observer reliability for HU $_{tumor}$, HU $_{intestinal\ wall}$ and SD $_{muscle}$.

	Interobserver Agreement (ICC, 95%CI)					
	HU $_{tumor}$		HU $_{intestinal\ wall}$		SD $_{muscle}$	
	Arterial Phase	Venous Phase	Arterial Phase	Venous Phase	Arterial Phase	Venous Phase
**40 keV**	0.949 (0.688-0.992)	0.900 (0.673-0.972)	0.953 (0.625-0.995)	0.951 (0.713-0.991)	0.909 (0.634-0.977)	0.927 (0.773-0.976)
**50 keV**	0.953 (0.704-0.993)	0.901 (0.056-0.989)	0.954 (0.637-0.995)	0.955 (0.738-0.992)	0.902 (0.303-0.986)	0.923 (0.732-0.977)
**60 keV**	0.960 (0.746-0.994)	0.901 (0.058-0.989)	0.967 (0.723-0.996)	0.964 (0.768-0.994)	0.928 (0.584-0.987)	0.915 (0.625-0.981)
**70 keV**	0.958 (0.738-0.994)	0.908 (0.116-0.990)	0.984 (0.850-0.998)	0.976 (0.845-0.996)	0.930 (0.597-0.988)	0.915 (0.627-0.981)
**80 keV**	0.946 (0.700-0.992)	0.938 (0.411-0.993)	0.986 (0.879-0.998)	0.978 (0.791-0.997)	0.934 (0.610-0.990)	0.915 (0.624-0.980)
**90 keV**	0.947 (0.679-0.992)	0.929 (0.321-0.992)	0.988 (0.890-0.998)	0.987 (0.884-0.998)	0.958 (0.706-0.994)	0.913 (0.616-0.980)
**100 keV**	0.943 (0.656-0.991)	0.966 (0.717-0.996)	0.988 (0.890-0.998)	0.993 (0.939-0.999)	0.964 (0.661-0.996)	0.913 (0.566-0.982)
**110 keV**	0.943 (0.655-0.991)	0.966 (0.720-0.996)	0.993 (0.939-0.999)	0.979 (0.802-0.997)	0.965 (0.671-0.996)	0.909 (0.599-0.979)
**120 keV**	0.938 (0.630-0.991)	0.967 (0.722-0.996)	0.992 (0.933-0.999)	0.980 (0.808-0.997)	0.960 (0.717-0.994)	0.909 (0.600-0.979)
**130 keV**	0.921 (0.547-0.988)	0.968 (0.732-0.996)	0.994 (0.949-0.999)	0.975 (0.762-0.997)	0.961 (0.723-0.994)	0.910 (0.602-0.979)
**140 keV**	0.928 (0.5830.989)	0.965 (0.711-0.996)	0.995 (0.961-0.999)	0.973 (0.749-0.997)	0.961 (0.727-0.994)	0.904 (0.522-0.981)
**150 keV**	0.914 (0.513-0.987)	0.964 (0.700-0.996)	0.994 (0.949-0.999)	0.974 (0.755-0.997)	0.957 (0.893-0.983)	0.937 (0.529-0.993)
**160 keV**	0.943 (0.6510.991)	0.963 (0.694-0.996)	0.992 (0.925-0.999)	0.972 (0.736-0.997)	0.904 (0.520-0.980)	0.932 (0.598-0.990)
**170 keV**	0.924 (0.560-0.989)	0.966 (0.718-0.996)	0.995 (0.961-0.999)	0.960 (0.619-0.995)	0.931 (0.655-0.986)	0.926 (0.464-0.992)
**180 keV**	0.933 (0.603-0.990)	0.966 (0.718-0.9960	0.994 (0.950-0.999)	0.969 (0.706-0.996)	0.945 (0.578-0.994)	0.936 (0.618-0.990)
**190 keV**	0.913 (0.510-0.987)	0.964 (0.706-0.996)	0.994 (0.950-0.999)	0.965 (0.671-0.996)	0.957 (0.697-0.994)	0.929 (0.648-0.987)
**200 keV**	0.916 (0.522-0.987)	0.963 (0.697-0.996)	0.993 (0.934-0.999)	0.965 (0.668-0.996)	0.919 (0.535-0.988)	0.929 (0.648-0.987)
**PEI**	0.967 (0.783-0.995)	0.957 (0.654-0.995)	0.963 (0.763-0.994)	0.961 (0.629-0.996)	0.952 (0.701-0.993)	0.951 (0.535-0.995)

ICC: interclass correction coefficient; 95%CI: 95% confidence interval; HU: hounsfield unit; SD: standard deviation; PEI: polyenergetic image.

Table S3. Objective image quality analysis.

	Image noise		Tumor SNR		Tumor CNR		Tumor Contrast	
	Arterial Phase	Venous Phase	Arterial Phase	Venous Phase	Arterial Phase	Venous Phase	Arterial Phase	Venous Phase
**40 keV**	11.05 (9.70,12.95)	11.10 (10.00,12.55)*	15.56 (13.93,18.00)*	19.21 (16.25,23.00)*	10.71 (8.34,13.00)*	12.00 (8.62,15.69)*	82.00 (61.00,125.0)*	133.0 (96.90,173.8)*
**50 keV**	10.60 (9.30,12.40)*	10.85 (9.95,12.20)	10.48 (8.43,11.97)	14.42 (11.74,16.55)	5.99 (3.77,8.00)	7.45 (5.24,9.25)	81.80 (60.50,124.0)*	72.90 (62.50,99.40)
**60 keV**	10.50 (9.00,12.35)	10.75 (9.90,11.95)	8.70 (6.76,9.78)	11.15 (9.42,12.29)	4..03 (2.65,6.36)	7.21 (5.69,8.77)	56.60 (45.20,86.50)	78.70 (56.50,95.30)
**70 keV**	10.40 (8.95,12.40)	10.60 (9.75,11.85)	7.95 (5.69,8.79)	9.14 (7.77,10.21)	3.57 (2.18,5.37)	6.25 (5.32,7.17)	56.60 (41.00,86.20)	66.60 (49.70, 78.10)
**80 keV**	10.35 (8.95,12.40)	10.55 (9.60,11.80)	7.03 (5.02,7.87)	7.92 (6.72,8.62)	3.19 (1.93,4.70)	5.41 (5.38,6.58)	47.60 (35.00,65.70)	58.70 (43.90,69.70)
**90 keV**	10.25 (8.95,12.40)	10.45 (9.60,11.70)	6.42 (4.56,7.31)	7.15 (6.10,7.87)	3.08 (1.74,4.22)	5.02 (5.78,6.17)	37.20 (29.00,57.00)	53.00 (40.90,64.40)
**100 keV**	10.35 (8.80,12.40)	10.50 (9.70,11.85)	5.96 (4.39,6.94)	6.47 (5.68,7.28)	2.92 (1.52,3.84)	4.77 (3.42,5.54)	36.50 (25.60,47.80)	51.70 (38.30,57.90)
**110 keV**	10.20 (8.80,12.35)	10.45 (9.95,11.75)	5.66 (4.18,6.60)	5.93 (5.40,6.89)	2.89 (1.33,3.56)	4.46 (3.14,5.24)	33.00 (21.90,44.60)	49.30 (36.60,53.80)
**120 keV**	10.20 (8.90,12.45)	10.50 (9.70,11.75)	5.43 (4.02,6.45)	5.61 (5.16,6.51)	2.83 (1.20,3.31)	4.31 (2.87,4.87)	26.70 (19.20,42.80)	46.60 (35.00,50.90)
**130 keV**	10.25 (8.90,12.35)	10.50 (9.55,11.60)	5.26 (3.91,6.30)	5.38 (4.92,6.40)	2.81 (1.17,3.12)	4.16 (2.74,4.79)	25.70 (16.90,40.90)	45.10 (32.80,49.90)
**140 keV**	10.20 (8.90,12.40)	10.50 (9.55,11.65)	5.12 (3.85,6.15)	5.18 (4.81,6.24)	2.72 (1.09,3.01)	4.05 (2.59,4.73)	24.40 (15.70,40.90)	43.70 (31.00,49.00)
**150 keV**	10.20 (8.80,12.40)	10.50 (9.55,11.75)	5.04 (3.77,5.82)	4.96 (4.66,6.13)	2.72 (0.99,2.99)	4.06 (2.50,4.66)	24.00 (14.90,38.50)	43.50 (30.00,48.80)
**160 keV**	10.50 (9.00,11.95)	10.50 (9.55,11.70)	4.58 (3.75,5.84)	4.83 (4.53,5.99)	2.55 (0.95,2.91)	3.95 (2.46,4.66)	23.60 (14.70,37.00)	41.60 (28.80,48.90)
**170 keV**	10.20 (8.80,12.50)	10.55 (9.60,11.60)	4.87 (3.66,5.95)	4.77 (4.43,5.91)	2.60 (0.96,2.94)	3.87 (2.46,4.66)	23.60 (13.60,37.10)	41.60 (28.10,49.20)
**180 keV**	10.20 (8.80,12.45)	10.50 (9.55,11.60)	4.82 (3.61,5.90)	4.68 (4.33,5.87)	1.76 (0.91,2.73)	3.85 (2.42,4.62)	23.00 (12.80,36.40)	40.60 (27.90,48.50)
**190 keV**	10.20 (8.80,12.40)	10.40 (9.50,11.60)	4.73 (3.56,5.87)	4.61 (4.25,5.82)	2.60 (0.88,2.82)	3.81 (2.40,4.67)	22.20 (13.10,36.20)	39.60 (27.60,47.90)
**200 keV**	10.10 (8.80,12.40)	10.40 (9.50,11.60)	4.68 (3.52,5.87)	4.56 (4.21,5.79)	2.53 (0.86,2.91)	3.77 (2.39,4.74)	22.60 (12.40,35.90)	40.50 (27.00,48.60)
**PEI**	12.30 (10.90,14.74)	12.75 (11.65,14.75)	6.00 (5.31,7.82)	7.62 (6.33,8.61)	2.97 (1.00,4.42)	5.01 (3.64,6.24)	22.30 (12.10,35.80)	61.99 (48.81,75.40)

Note: Data are expressed as median (interquartile ranges).

SNR: signal-to-noise ratio; CNR: contrast-to-noise ratio; PEI: polyenergetic image.

* indicate significant differences ($p < 0.05$) of VMI 40–200 keV compared to PEI.
